# Eccentricity Fault Diagnosis System in Three-Phase Permanent Magnet Synchronous Motor (PMSM) Based on the Deep Learning Approach

**DOI:** 10.3390/s25247416

**Published:** 2025-12-05

**Authors:** Kenny Sau Kang Chu, Kuew Wai Chew, Yoong Choon Chang, Stella Morris, Yap Hoon, Chen Chen

**Affiliations:** 1 Lee Kong Chian Faculty of Engineering and Science, Universiti Tunku Abdul Rahman, Kajang 43200, Malaysia; kennysaukangchu@gmail.com (K.S.K.C.); ycchang@utar.edu.my (Y.C.C.); stellam@utar.edu.my (S.M.); hoonyap@utar.edu.my (Y.H.); 2 School of Telecommunications Engineering, Xidian University, Xi’an 710071, China; cc2000@mail.xidian.edu.cn; 3 Xidian Guangzhou Institute of Technology, Xidian University, Guangzhou 510555, China

**Keywords:** motor eccentricity fault, fault diagnosis system, neural network

## Abstract

Motor eccentricity faults, stemming from the misalignment of the rotor’s center and pivot point, lead to significant vibrations and noise, compromising motor reliability. This study emphasizes the need for an efficient diagnostic system to enable early detection and correction of these faults. Our research proposes a novel Eccentricity Fault Diagnosis Network (E-FDNet), designed for integration into a Motor Eccentricity Fault Diagnosis System (MEFDS), utilizing neural networks for detection. Evaluation tests reveal that a hybrid Convolutional Neural Network-Long Short-Term Memory (CNN-LSTM) architecture is ideal as the internal neural network within the E-FDNet. Key contributions of this research include (1) E-FDNet that stabilizes transition predictions among SEF/DEF/MEF; (2) a steady-state characteristic normalization (SSCN) improving feature consistency under dynamic responses; (3) an integrated physics–FEM–experiment pipeline for controlled analysis and validation; (4) approximately 98.86% accuracy/F1 outperforming classical and deep baselines; and (5) a non-invasive, current-only sensing design suited for deployment.

## 1. Introduction

Permanent Magnet Synchronous Motors (PMSMs) are widely used in the electric automotive industry due to their high efficiency and excellent torque characteristics. The mechanical integrity of the motor is a crucial factor in ensuring the system performs reliably. Motor faults have become a key concern for researchers, as mechanical faults are common in motor systems. Various types of mechanical faults can occur, including stator inter-turn winding faults, bearing faults, and eccentricity faults.

This paper focuses on eccentricity faults, which occur when the rotor’s pivot point and center deviate from their intended alignment. When an eccentricity fault occurs, it can lead to motor vibrations and noise. Therefore, developing an effective eccentricity fault diagnosis system is essential for reducing eccentricity and improving motor health. The primary causes of eccentricity faults include wear and tear, mechanical defects, and overloading. There are three main types of eccentricity faults: (1) Static Eccentricity Fault (SEF): The rotor is displaced from its normal center position, but the axis of rotation remains fixed. This results in an uneven air gap, but the magnitude remains constant due to the fixed rotor position [1]. (2) Dynamic Eccentricity Fault (DEF): The rotor’s axis of rotation is not fixed and varies as the motor rotates, causing a fluctuating air gap between the rotor and the stator [2]. (3) Mixed Eccentricity Fault (MEF): A combined static-dynamic eccentricity fault condition [3].

A data-driven approach leverages data analysis and interpretation to make predictions or decisions. To improve the accuracy of fault diagnosis systems, many researchers have integrated traditional methods with machine learning techniques [4]. For instance, in [5], Motor Current Signature Analysis (MCSA) was used to extract detailed parameters from the current signal. Principal Component Analysis (PCA) was then applied for dimensionality reduction, followed by K-Nearest Neighbors (KNN) as a classifier to identify the type of eccentricity fault. This model successfully detected SEF and DEF, even in the presence of noise. Similarly, in [1], a model was proposed to detect SEF using the incremental inductance curve of the motor’s d-axis. The saturation current and the height of the inductance curve hump were used as inputs for KNN, which was trained to classify the system’s condition. Another study [6] introduced Topological Data Analysis (TDA) in MCSA applications for eccentricity fault detection. TDA was employed to extract topological features, which were then used to construct a persistence diagram and convert it into Betti curves for KNN training. The results demonstrated that TDA effectively identified SEF. In [7], both MCSA and torque analysis were used to collect data, which was then processed using Classification and Regression Trees (CART), Support Vector Machines (SVM), and KNN. Among these models, SVM showed the best performance in detecting faults (both end-of-line test faults and eccentricity faults). A novel Concept Drift Detector was introduced in [8], utilizing Typicality and Eccentricity Data Analytics (TEDA). The model classified faults using concept drift methods, effectively distinguishing between different fault types.

Neural Networks (NNs) play a crucial role in fault diagnosis by enhancing data analysis and classification accuracy. These models can automatically extract key features from data, making them highly effective for identifying system conditions. For example, in [9], a Backpropagation (BP) Neural Network was trained on mixed eccentricity data from a parametric analytical model of back electromotive force (EMF). The model, trained on 34,420 data samples, achieved high precision in detecting MEF. In [10], an Autoencoder (AE) was used as a noise reconstruction model to improve the signal-to-noise ratio. A Feature Extraction and Classification (FEC) model was then developed as a multi-layer network to detect faults from stator current signals. The proposed method achieved an average accuracy of 90.26% ± 1.18% for bearing fault detection and 96.25% ± 0.63% for eccentricity fault detection. A Physics-Informed Adversarial Network was introduced in [11], utilizing a Generative Adversarial Network (GAN) trained on data from experimental setups (normal conditions), a physics-informed mathematical model (all conditions), and the Finite Element Method (FEM) (normal conditions only). The model was validated using FEM fault data, with Mean Absolute Error (MAE) used as the evaluation metric. However, the model could not classify the specific type of eccentricity fault. In [12], a GAN was trained on EMF signal data under normal conditions and SEF, using mathematical modeling and real data. Mean Absolute Percentage Error (MAPE) was employed as a performance metric, with a penalty term added to enhance training efficiency. An improved GAN for vibration signal-based fault detection was proposed in [13]. The model classified SEF, rotor broken bar faults, and their combinations using a Softmax function. A hybrid Deep Convolutional Neural Network (CNN) and Support Vector Machine (SVM) model was introduced in [14] to detect multiple faults. The CNN extracted features, while PCA reduced dimensionality. The SVM classifier then classified misalignment, unbalance, and rotor disk eccentricity as single, dual, and multi-faults. The model was trained on data from Infrared Thermography (IRT) and vibration signals. In [15], a hybrid model combining ConvNeXt, ResNet, CNN, and GCNet (CRCG) was proposed for eccentricity fault detection. The model was trained on 2D images obtained by converting 1D magnetic flux density signals using Gramian Angular Field (GAF) and Markov Transition Field (MTF) techniques. The model achieved an accuracy of 97.4% and an F1-score of 97.9%.

The exceedingly narrow air gap in industrial motors presents a significant challenge, making fault-induced signal changes subtle and difficult to detect. Current research frequently lacks comprehensive classification of Static, Dynamic, and Mixed Eccentricity Faults (SEF, DEF, and MEF), as many diagnostic methods are specialized for particular fault types. This often necessitates deploying multiple models to cover all fault conditions, consequently imposing a considerable computational burden. Furthermore, most datasets used for training are limited to steady-state operating conditions, reducing the models’ ability to generalize to dynamic or transitional fault scenarios. The key contributions of this paper are as follows:

1. **Eccentricity Fault Diagnosis Network (E-FDNet):** A framework (CNN–LSTM Network) that stabilizes transition predictions and resolves class ambiguity among SEF/DEF/MEF.
2. **Steady-State Characteristic Normalization (SSCN):** An automatic scheme that finds steady-state segments and scales by a characteristic amplitude, improving feature consistency under dynamic responses and reducing training instability.
3. **Physics–simulation–experiment pipeline:** A physics-based PMSM eccentricity model and FEM setup integrated with hardware-acquired normal data, enabling controlled analysis and validation of SEF, DEF, and MEF.
4. **End-to-end performance gains:** Within one E-FDNet pipeline, the CNN–LSTM backbone attains ≈98% accuracy and ≈98% F1 with short detection latency (about 1–3 ms)
5. **Non-invasive, current-only sensing:** Relies solely on stator current, avoiding extra sensors and supporting practical industrial deployment.

## 2. Mathematical Characteristics of Motor Eccentricity

When a PMSM operates under healthy conditions at constant speed, the stator current of each phase can be modeled as a single-frequency sinusoid at the electrical supply frequency ($f_{s}$). Under eccentricity faults, the non-uniform air gap introduces spatial asymmetry in the magnetic field, which in turn modulates the back-EMF and stator currents. This modulation generates additional spectral components around the fundamental and its harmonics, whose structure depends on the fault type (static, dynamic, or mixed). The phase stator current can be expressed as [16]:

(1) $$I_{\mathrm{normal}}\left(t\right)=I_{s}\cos\left(2\pi f_{s}t+\phi \right),$$

where $I_{s}$ is the peak stator current, $f_{s}$ is the electrical supply frequency, and ϕ is the phase angle. The mechanical rotational frequency is $f_{r}=\frac{f_{s}}{p}$, where *p* is the number of pole pairs.

Under eccentricity faults, spatial asymmetry in the air-gap produces modulation of the air-gap flux density and back-EMF, leading to additional components in the stator current spectrum. A convenient and dimensionally consistent representation of the fault current is

(2) $$I_{\mathrm{fault}}\left(t\right)=I_{s}\cos\left(2\pi f_{s}t+\phi \right)\;+\;\sum_{\nu =1}^{\infty }\sum_{m=0}^{\infty }I_{\nu ,m}\;\cos\;\left(2\pi \;\left(\nu f_{s}\pm mf_{r}\right)\;t+\phi_{\nu ,m}\right),$$

where ν denotes the electrical space-harmonic order excited by the winding distribution and slotting effects, while *m* is the modulation (sideband) index associated with the mechanical rotational frequency. The coefficients ($I_{\nu ,m}$) and phases ($\phi_{\nu ,m}$) describe the amplitude and phase of each sideband component, respectively. Together, these terms capture how air-gap asymmetry redistributes energy from the fundamental into sidebands at frequencies

### 2.1. Static Eccentricity (SEF)

In SEF, the rotor center is offset, and the minimum air-gap position is fixed in the stator reference frame. The air gap is non-uniform in space but does not rotate past the stator teeth. Consequently, SEF primarily excites or modifies the amplitudes of integer multiples of the electrical fundamental without introducing time-varying sidebands:

(3) $$f_{\mathrm{SEF}}\in \left\{\;\nu f_{s}\;|\;\nu =1,2,3,\ldots \;\right\}.$$

(Practically, ν=1 and low-order harmonics dominate; which ν are significant depends on winding distribution and inherent slotting/space harmonics.)

### 2.2. Dynamic Eccentricity (DEF)

In DEF, the minimum air-gap position is locked to the rotor and rotates past stator teeth. This produces amplitude/phase modulation at $f_{r}$ (and its multiples), generating sidebands around $\nu f_{s}$:

(4) $$f_{\mathrm{DEF}}\in \left\{\;|\nu f_{s}\pm mf_{r}|\;|\;\nu =1,2,3,\ldots ;\;m=1,2,3,\ldots \right\}.$$

### 2.3. Mixed Eccentricity (MEF)

MEF combines SEF and DEF, so both the carrier components and their rotational sidebands appear:

(5) $$f_{\mathrm{MEF}}\in \left\{\;\nu f_{s}\;\right\}\;\cup \;\{\;|\nu f_{s}\pm mf_{r}\left|\;\right\}.$$

Correspondingly, the time-domain current can be written as

(6) $$I_{\mathrm{MEF}}\left(t\right)=I_{s}\cos\left(2\pi f_{s}t+\phi \right)+\sum_{\nu }I_{\nu ,0}\cos\;\left(2\pi \;\nu f_{s}\;t+\phi_{\nu ,0}\right)+\sum_{\nu }\sum_{m\geq 1}I_{\nu ,m}\cos\;\left(2\pi \;\left(\nu f_{s}\pm mf_{r}\right)\;t+\phi_{\nu ,m}\right).$$

## 3. Methodology

### 3.1. Experimental Setup

All simulations and training were conducted on an Acer Nitro laptop (Acer Inc., Taipei, Taiwan) equipped with an Intel Core i5-2050 processor and an NVIDIA RTX GPU, running Windows 11. The implementation was developed in Python 3.12 using TensorFlow and Keras 3. Stator current signals were acquired using a Hantek 6074BC USB oscilloscope at a sampling rate of approximately 20.5 kHz. The data were segmented into windows of 512 samples. Training was performed with a batch size of 64 using the Adam optimizer (learning rate = 0.001) for 500 epochs, with early stopping enabled.

**Figure 1:**
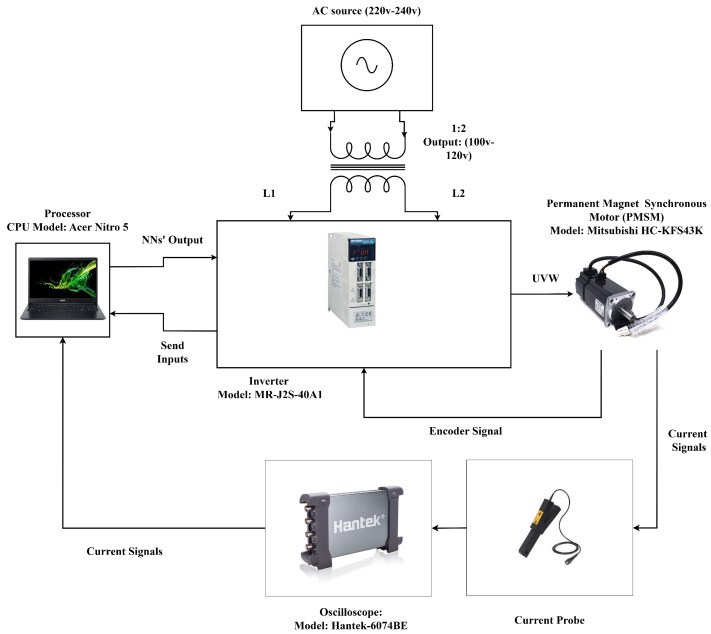
Block diagram of the experimental setup.

**Figure 2:**
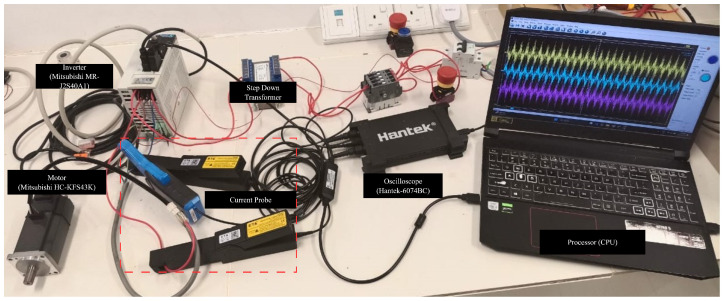
Experimental setup.

In this experiment, a Mitsubishi HC-KFS43 motor (Mitsubishi Electric Corporation, Tokyo, Japan) was employed to validate the proposed method. The motor specifications are provided in Table 1. Figure 1 illustrates the block diagram of the experimental setup, while Figure 2 depicts the physical configuration. An oscilloscope was utilized to capture the motor phase currents, which were subsequently transmitted to the CPU. The motor was driven by an MR-J2S-40A1 inverter (Mitsubishi Electric Corporation, Tokyo, Japan) that both controlled the motor and measured the rotational speed. The inverter transferred the data to the CPU, where they were processed and analyzed by the trained neural network (NN) for fault detection. Since the motor operates within a voltage range of 100–120 V, a transformer was used to step down the AC power supply from 220–240 V.

**Figure 3:**
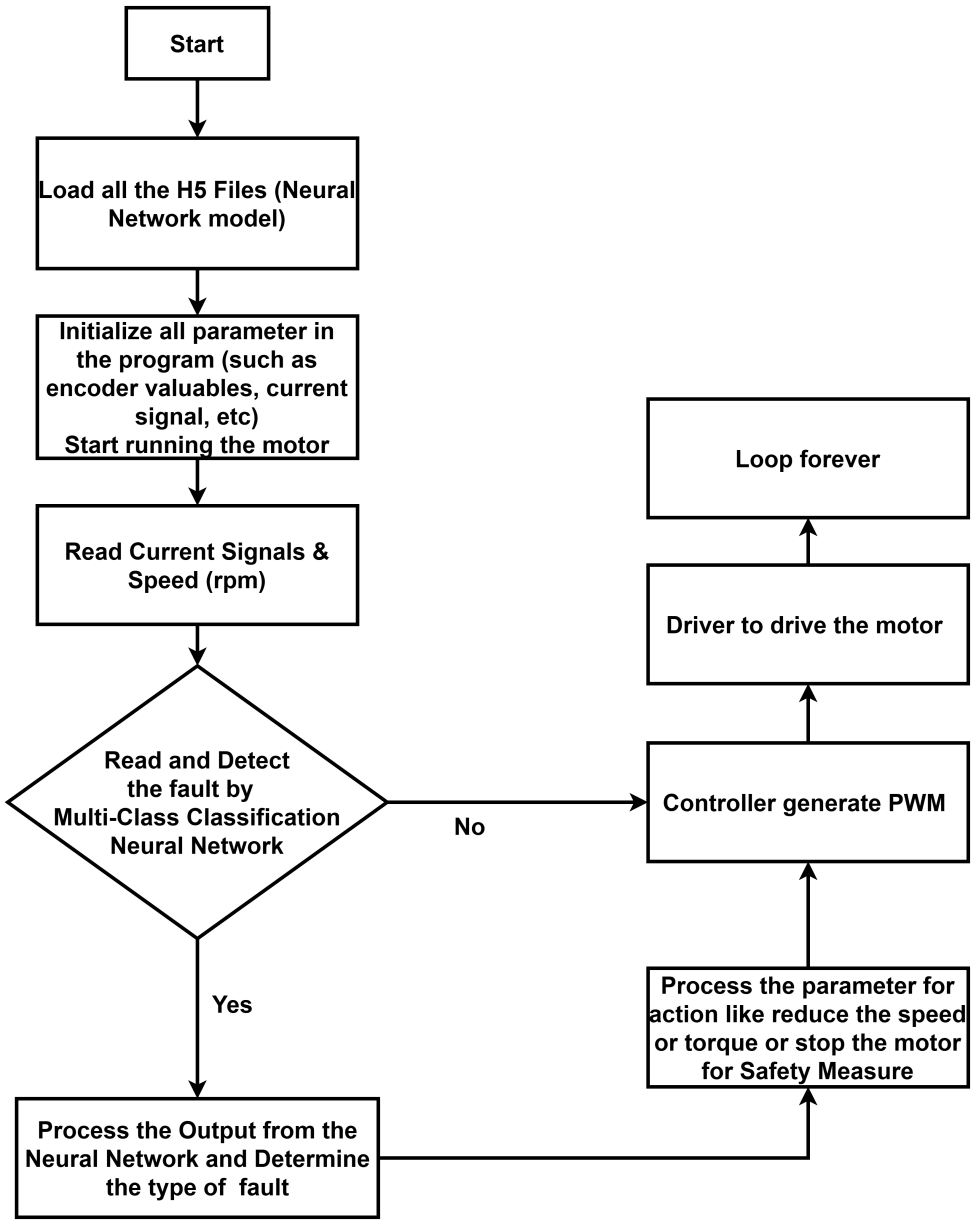
Flow chart of the MEFDS.

Figure 3 illustrates the flowchart of the MEFDS, which represents the fault diagnosis process. At the beginning of the program, the neural network (NN) model is loaded, and all parameters are initialized. During motor operation, the speed signal is acquired from the encoder, while the current signals are obtained from the oscilloscope. The NN model processes these signals to extract relevant features for fault detection. If the NN output indicates normal operation, the system continues running without interruption. Conversely, if a fault is detected, the processor sends a signal to the controller to indicate the fault and stop the motor. The program operates in a continuous loop.

### 3.2. Data Preprocessing and Labelling

Developing a dataset that includes all conditions is crucial to ensuring the accuracy of the system. Common methods for acquiring signals from different conditions include experimental methods, simulation methods such as the finite-element method (FEM), and physics-based mathematical models (PMM).

The experimental method is the most realistic and accurate approach, but it is also the most costly and time-consuming [11,18]. This is because obtaining data for normal and three faulty conditions requires at least four motors, making it difficult to generate a large dataset with various parameter combinations. A small dataset can only cover a limited range of conditions, reducing the adaptability of the diagnosis system. In [19], MCSA was used to diagnose SEF in PMSM. The stator current and back electromotive force (EMF) waveforms were analyzed to identify fault behaviour. However, the dataset was limited to SEF only, preventing the system from classifying DEF or MEF. In [20], a real-time monitoring diagnosis system was proposed to detect DEF using back EMF waveforms. Due to high costs, the experiment only generated DEF conditions ranging from 25% to 50%.

Simulation methods provide another approach to collecting data. A physics-based model is a traditional simulation technique that replicates system behaviour by solving motor equations. In [11], a physics-informed mathematical model of a PMSM was developed to generate SEF and DEF characteristic signals. FEM is another reliable technique for obtaining high-accuracy data. In [5], a time-stepping FEM was applied to a PMSM under SEF and DEF conditions. Wavelet decomposition was used to analyze stator currents, while principal component analysis (PCA) was employed for dimensionality reduction. A fuzzy support vector machine (FSVM) was then used as a classifier to identify the type of eccentricity fault.

In this research, both experimental and simulation data are combined. Table 2 presents the data used to construct the dataset for training the neural network, with the labelling scheme also shown in Table 2. The experimental dataset contains PMSM data under normal operating conditions. The simulation dataset consists of two subsets: one generated using the PMM method and the other obtained from the FEM model developed in ANSYS software (ANSYS 2024 r1). Table 3 summarizes the parameters used in the ANSYS simulations. Across these sources, the dataset covers Normal, SEF, DEF, and MEF conditions under different speeds and load levels, where higher loads naturally produce higher current amplitudes. In addition, controlled noise is injected into part of the simulated signals to emulate measurement disturbances and improve the robustness of the diagnostic model.

In the dynamic response of the system, the signals may exhibit inconsistencies, such as fluctuations in amplitude. This issue affects the training process, as the neural network cannot efficiently extract meaningful data characteristics [21].

In order to standardize the data, Steady-State Characteristic Normalization (SSCN) is employed in this experiment. This method is designed to identify the steady-state region of a signal and extract its characteristic value for normalization [22,23]. By ensuring that the extracted features remain consistent across different conditions, SSCN enhances the reliability and robustness of the data used for neural network training.

To detect the steady-state region, the method employs a rolling standard deviation analysis [24]. Given a current signal I(t), the rolling standard deviation is computed over a window of size *W* to assess the stability of the signal:

(7) $$\sigma_{\mathrm{rolling}}\left(t\right)=\sqrt{\frac{1}{W}\sum_{i=t-W+1}^{t}{\left(I\left(i\right)-\bar{I}\right)}^{2}}$$

where $\bar{I}$ represents the mean current over the given window.

The steady-state region is determined by identifying the first index $t_{s}$ at which the rolling standard deviation falls below a predefined threshold, set as a fraction of the maximum signal amplitude. The steady-state start index is obtained as:

(8) $$t_{s}=\min\left\{t|\sigma_{\mathrm{rolling}}\left(t\right)<0.05\max\left(I\right)\right\}$$

If no steady-state region is detected, the default start index is set to the midpoint of the signal to ensure a stable analysis.

Once the steady-state region is identified, the characteristic value of the signal is extracted. The maximum and minimum values within this steady-state segment are determined as:

(9) $$P_{\max}=\max I\left(t\right),\;P_{\min}=\min I\left(t\right),\;t\geq t_{s}$$

From these values, the characteristic amplitude is computed as:

(10) $$P_{\mathrm{char}}=\frac{P_{\max}+\left|P_{\min}\right|}{2}$$

After obtaining the characteristic amplitude, each value in the original signal is normalized by dividing it by $P_{\mathrm{char}}$, ensuring a consistent scale across different conditions:

(11) $$I_{\mathrm{norm}}\left(t\right)=\frac{I(t)}{P_{\mathrm{char}}},\;t\geq t_{s}$$

This normalization process enhances feature extraction by allowing the model to focus on dynamic variations rather than absolute magnitudes.

The SSCN method significantly enhances neural network performance by improving feature extraction and signal stability. By normalizing signals based on their steady-state characteristics, SSCN ensures that input features remain consistent, reducing sensitivity to variations in absolute magnitude. This stabilization prevents fluctuations from disrupting the training process, mitigates gradient-related issues, and improves overall convergence. Furthermore, SSCN enhances generalization across different operating conditions by ensuring that the model focuses on dynamic characteristics rather than absolute signal magnitudes. This enables neural networks, such as CNN-LSTM models, to better capture both temporal and spatial dependencies within the data. As a result, neural networks achieve higher accuracy in fault classification and anomaly detection for motor systems, leading to more reliable and interpretable diagnostics.

**Figure 4:**
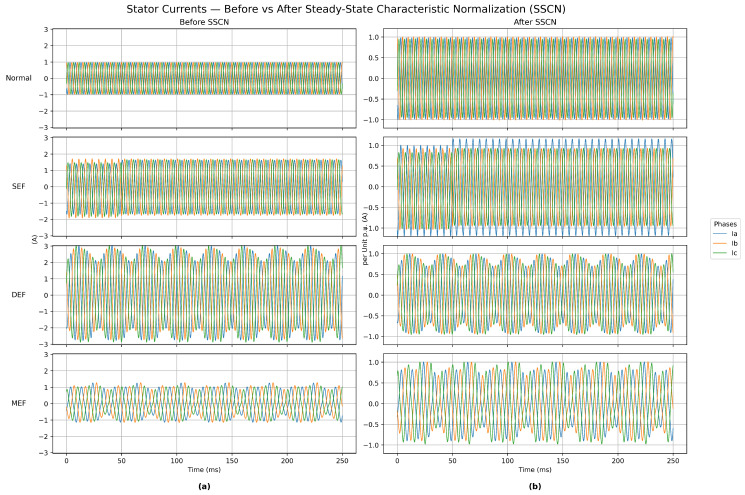
Stator current waveforms for four operating conditions (Normal, SEF, DEF, and MEF): (**a**) original three-phase currents before SSCN; (**b**) normalized three-phase currents after SSCN.

In the analysis of motor eccentricity, the same fault type can produce stator currents with different peak amplitudes under different speeds and loads, which complicates direct comparison and feature extraction. Figure 4 presents the three-phase stator current waveforms before and after the application of Steady-State Characteristic Normalization (SSCN) under four operating conditions: Normal, Static Eccentricity Fault (SEF), Dynamic Eccentricity Fault (DEF), and Mixed Eccentricity Fault (MEF), where (a) presents the original currents before SSCN and (b) shows the normalized currents after SSCN. By rescaling all current signals to a uniform per-unit amplitude range, SSCN enhances the comparability of data across fault types and operating states and thus improves the robustness of the learning process.

As shown in Figure 4, the Normal condition exhibits well-balanced sinusoidal waveforms typical of healthy operation. Under SEF, a vertical offset is observed, indicating the presence of a static air-gap asymmetry. In the DEF condition, periodic amplitude modulation appears, corresponding to time-varying eccentricity due to rotor displacement. The MEF condition demonstrates more irregular distortions, reflecting the combined effects of static and dynamic eccentricities. After applying SSCN, the signals are normalized to a consistent scale while these distinguishing temporal characteristics are preserved, which facilitates more effective feature extraction and enhances the neural network’s capability to distinguish between different eccentricity fault types.

### 3.3. Structure of the Eccentricity Fault Diagnosis Network (E-FDNet)

**Figure 5:**
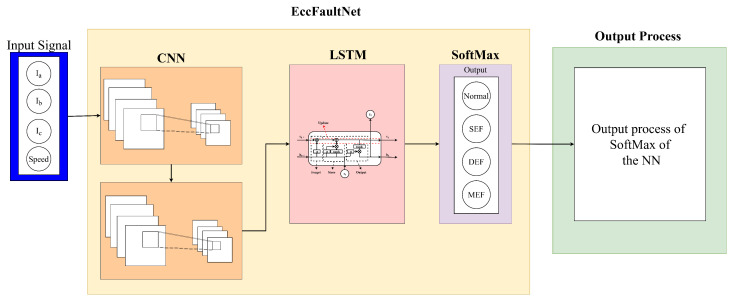
Architecture of Eccentricity Fault Diagnosis Network (E-FDNet).

After extensive experimentation with various neural network architectures, the proposed Eccentricity Fault Diagnosis Network (E-FDNet) adopts a hybrid one-dimensional Convolutional Neural Network and Long Short-Term Memory (CNN–LSTM) structure for accurate classification of four operating conditions of the Permanent Magnet Synchronous Motor (PMSM): Normal, Static Eccentricity Fault (SEF), Dynamic Eccentricity Fault (DEF), and Mixed Eccentricity Fault (MEF). The CNN layers are designed to extract localized temporal–spatial features from the three-phase current and reference speed signals, while the LSTM layer captures sequential dependencies over time, enabling the network to learn both transient and steady-state characteristics of motor behavior. The final fully connected and softmax layers perform the classification based on the learned feature representations. The overall architecture of the proposed E-FDNet is illustrated in Figure 5. The input dataset comprises four sequential features, namely the three-phase stator currents $\left(I_{a},I_{b},I_{c}\right)$ and the rotational speed, forming a time-series segment $X\in R^{T\times F}$, where T=500 represents the window length and F=4 the feature dimension. Each long recording is segmented into fixed-length windows using a sliding strategy with stride S=50, ensuring that no window crosses the boundary between different sample identities. The segmentation process can be expressed as

(12) $$X^{\left(n\right)}=\left[\begin{array}{c} x_{t}\\ x_{t+1}\\ \vdots \\ x_{t+W-1} \end{array}\right],\;y^{\left(n\right)}=y_{s},$$

where $y_{s}\in \left\{0,1,2,3\right\}$ denotes the class label associated with each sample *s* [25]. Prior to model training, all features are standardized using the statistics (mean $\mu_{f}$ and standard deviation $\sigma_{f}$) computed from the training subset, following

(13) $$x_{t,f}^{\prime }=\frac{x_{t,f}-\mu_{f}}{\sigma_{f}},$$

to ensure consistent numerical scaling across time-series channels [13].

The CNN–LSTM model combines convolutional feature extraction and temporal sequence modeling. The first stage consists of two one-dimensional convolutional blocks, each followed by batch normalization, ReLU activation, and max-pooling with a factor of two. These layers extract local temporal–spatial features and progressively reduce temporal resolution. The operation of a convolutional block can be described as

(14) $$H_{k}=f_{\mathrm{ReLU}}\;\left(\mathrm{BN}\;\left(\mathrm{Conv}1D(X;\;W_{k},b_{k})\right)\right),$$

where $W_{k}$ and $b_{k}$ denote the convolutional kernel and bias for the *k*-th block, and BN represents batch normalization. The feature sequence after the second convolutional block, $H_{2}\in R^{T^{\prime }\times D}$, where $T^{\prime }=T/4$, is passed to an LSTM unit to capture long-range dependencies across time [26]. The LSTM cell updates its hidden and cell states through

(15) $$\begin{array}{cc} i_{t} & =\sigma \left(W_{i}u_{t}+U_{i}h_{t-1}+b_{i}\right),\;f_{t}=\sigma \left(W_{f}u_{t}+U_{f}h_{t-1}+b_{f}\right),\\ o_{t} & =\sigma \left(W_{o}u_{t}+U_{o}h_{t-1}+b_{o}\right),\;{\tilde{c}}_{t}=\tanh\left(W_{c}u_{t}+U_{c}h_{t-1}+b_{c}\right),\\ c_{t} & =f_{t}⊙c_{t-1}+i_{t}⊙{\tilde{c}}_{t},\;h_{t}=o_{t}⊙\tanh\left(c_{t}\right), \end{array}$$

where σ(·) and tanh(·) denote the sigmoid and hyperbolic tangent functions, respectively. The final hidden state $h_{T}$ serves as a compact temporal representation of the windowed signal [27].

Subsequently, a fully connected layer with ReLU activation refines this temporal representation as

(16) $$z=f_{\mathrm{ReLU}}\left(W_{d}h_{T}+b_{d}\right),$$

which is then passed to a softmax output layer for multiclass classification across C=4 classes:

(17) $$\hat{y}=\mathrm{softmax}\left(W_{o}z+b_{o}\right).$$

The softmax function converts the logits into normalized probabilities such that

(18) $${\hat{y}}_{i}=\frac{e^{z_{i}}}{\sum_{j=1}^{C}e^{z_{j}}},$$

where ${\hat{y}}_{i}$ is the predicted probability corresponding to class *i* [28,29]. The model is trained using the categorical cross-entropy loss function:

(19) $$L=-\frac{1}{N}\sum_{n=1}^{N}\sum_{i=1}^{C}y_{n,i}\log\left({\hat{y}}_{n,i}\right),$$

where $y_{n,i}$ denotes the one-hot encoded ground truth of the *n*-th sample. The Adam optimizer with a learning rate of $10^{-3}$ is utilized to minimize the loss, and the training process employs early stopping (patience = 20) with learning rate reduction (factor = 0.5) to enhance convergence stability. The optimal model weights are selected based on the lowest validation loss [30]. Finally, the predicted class label for each window is obtained as

(20) $$\tilde{y}=\arg\max_{i}\left({\hat{y}}_{i}\right),$$

and overall performance is evaluated using accuracy, macro- and micro-averaged F1-scores, and a normalized confusion matrix defined by

(21) $${\tilde{C}}_{i,j}=\frac{C_{i,j}}{\sum_{k=1}^{C}C_{i,k}},$$

where $C_{i,j}$ denotes the number of samples belonging to class *i* that are predicted as class *j*. This hybrid CNN–LSTM framework effectively integrates local feature extraction and temporal dependency modeling, providing a robust and accurate approach for PMSM fault classification [31].

## 4. Result & Discussion

**Figure 6:**
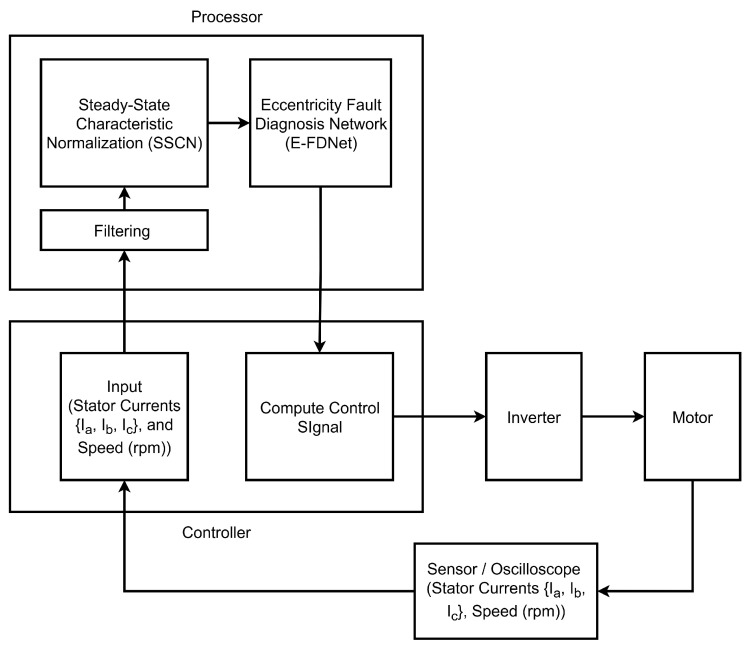
General structure of the motor system.

Figure 6 illustrates the general structure of the motor system. The stator current signals are acquired using a sensor or an oscilloscope and then transmitted to the controller, which subsequently forwards them to the processor. The motor speed is measured by an encoder. Initially, the raw stator current signals undergo filtering to reduce noise, followed by normalization using the SSCN. After normalization, the current signals and speed data are processed by the E-FDNet to predict the state of the motor system. If no fault is detected, the controller sends a pulse-width modulation (PWM) signal to the driver for normal operation. Conversely, if a fault is detected, the controller adjusts the PWM signal to either reduce the motor speed or stop the motor entirely.

### 4.1. Evaluation of Performance Under Different Types of Neural Networks

In the proposed E-FDNet framework, selecting an appropriate neural network architecture is crucial. Since this research involves a classification task with an imbalanced dataset (with more fault data than normal operating data), the F1-score is chosen as the primary evaluation metric. The F1-score is well suited for such scenarios because it provides a robust measure of classification performance by balancing precision and recall [32].

To conduct a fair and representative comparison within the E-FDNet framework, several neural network architectures were selected to cover the main families used in time-series classification and fault diagnosis. A fully connected Deep Neural Network (DNN) is included as a non-convolutional baseline. A one-dimensional convolutional neural network (CNN) represents convolution-based feature extractors that capture local temporal patterns, while the Temporal Convolutional Network (TCN) extends this idea using deeper, causal convolutions with dilation. A Transformer-based model is considered as an attention-driven architecture capable of modeling global dependencies. Finally, the proposed hybrid CNN–LSTM combines convolutional layers with recurrent units to jointly exploit spatial and temporal information in the stator current signals. The hyperparameter configurations for each network are listed in Table 4.

Table 5 summarizes the corresponding performance metrics of the tested neural network models within the Eccentricity Fault Diagnosis Network (E-FDNet) framework. Among the evaluated models, the CNN–LSTM architecture demonstrates the best overall performance, achieving the highest classification accuracy across all fault categories and attaining an average F1-score of 98.86%. The CNN model also performs competitively, with an overall F1-score of 95.31%, showing particularly strong accuracy in detecting Label 0 (Normal) and Label 2 (Dynamic Eccentricity Fault), both exceeding 99%. Similarly, the Temporal Convolutional Network (TCN) yields a comparable accuracy of 95.08%, confirming that both CNN-based architectures are effective in capturing spatio-temporal fault patterns.

However, both CNN and TCN exhibit a moderate drop (around 10%) in the detection accuracy for Label 1 (Static Eccentricity Fault) and Label 3 (Mixed Eccentricity Fault), with accuracy values near 90%. In contrast, the Deep Neural Network (DNN) achieves a lower F1-score of 82.00%, showing strong performance for Label 0 (≈92%) but relatively weak recognition for Label 1 (≈68%) and Label 3 (≈75%). The Transformer-based model produces an F1-score of 80.09%, with moderate accuracy (≈90%) for the normal class but limited performance (≈70–76%) for the fault classes. Overall, the hybrid CNN–LSTM structure exhibits the most robust generalization and stability across all fault categories, confirming its superior capability in capturing both spatial and temporal dependencies inherent in PMSM eccentricity fault signals. From a computational perspective, the CNN–LSTM has the highest complexity among the tested models, as it combines convolutional blocks with an LSTM layer and a fully connected classification head. However, for the input length used in this study (T=500 samples and F=4 features), the end-to-end detection latency of E-FDNet (including preprocessing and inference) is only about 0.001–0.003 s. This duration is substantially shorter than the 20 ms diagnostic window and is well within real-time protection requirements for the PMSM drive.

**Figure 7:**
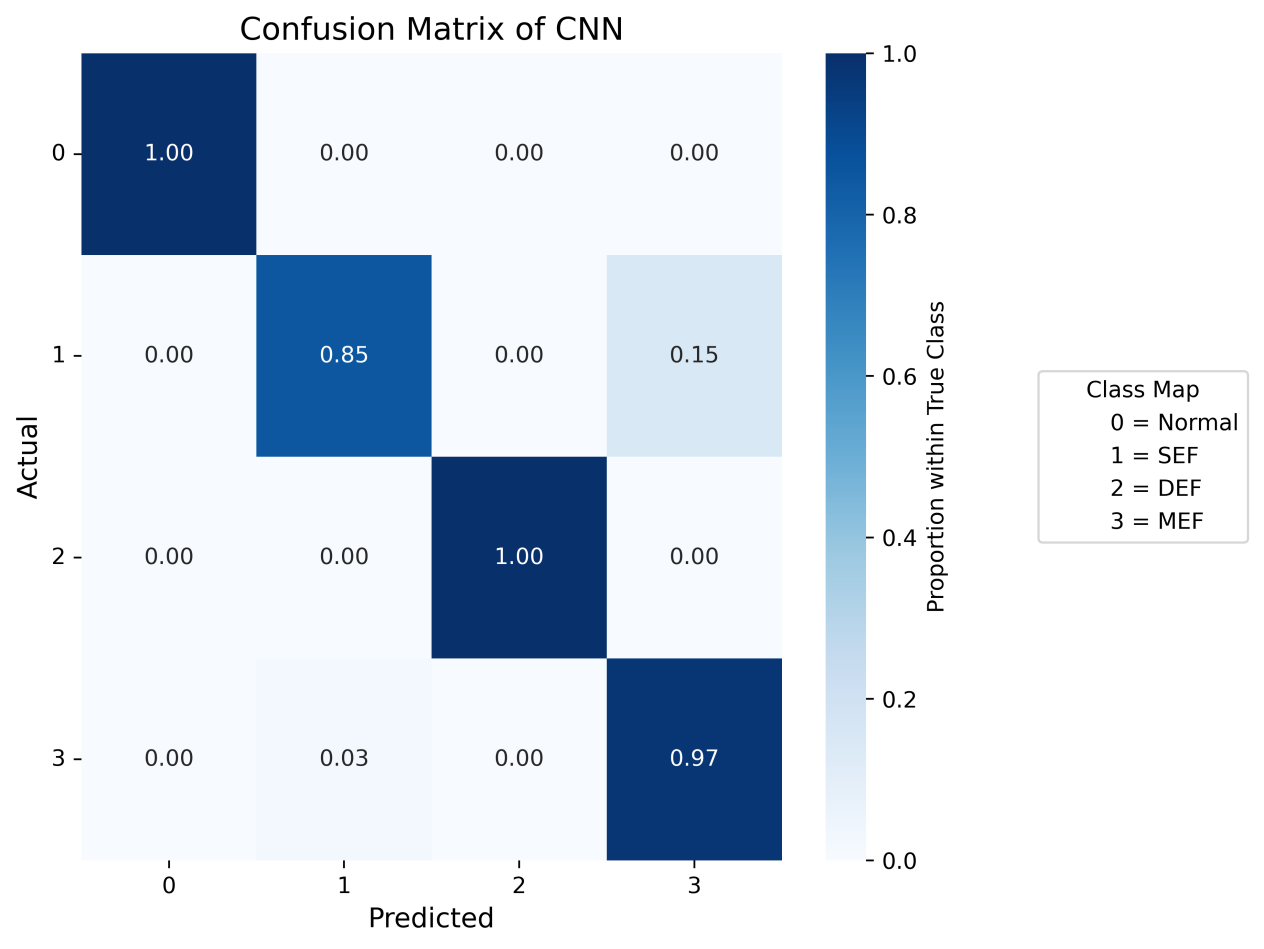
Confusion matrix of CNN.

**Figure 8:**
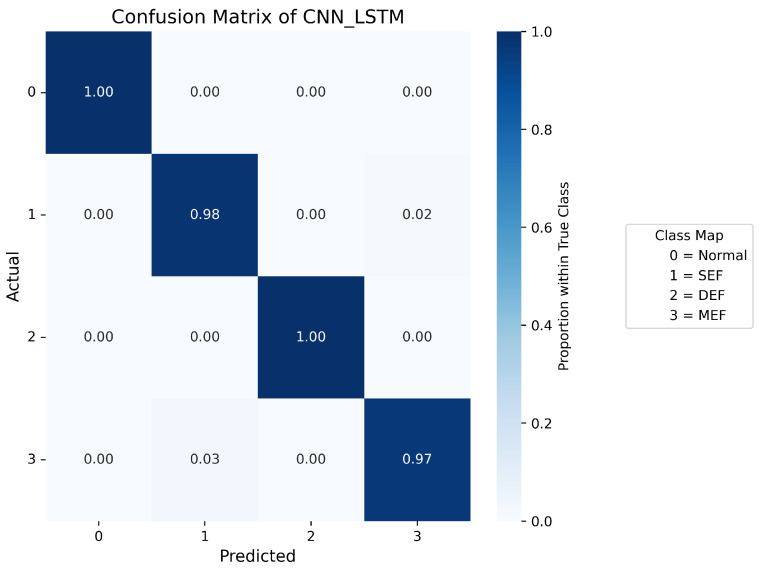
Confusion matrix of CNN-LSTM.

**Figure 9:**
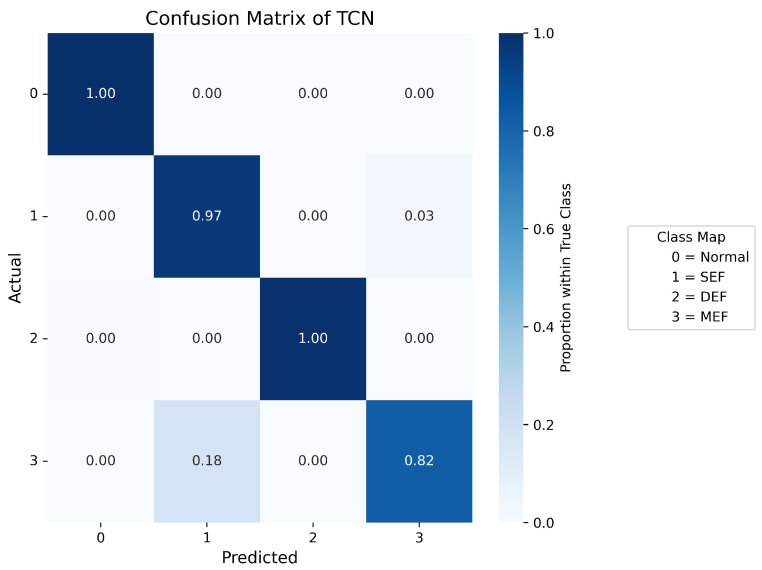
Confusion matrix of TCN.

**Figure 10:**
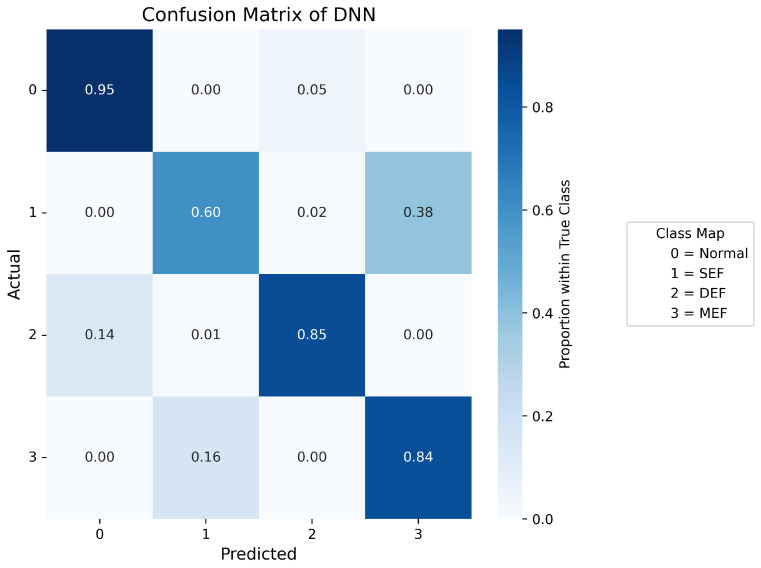
Confusion matrix of DNN.

**Figure 11:**
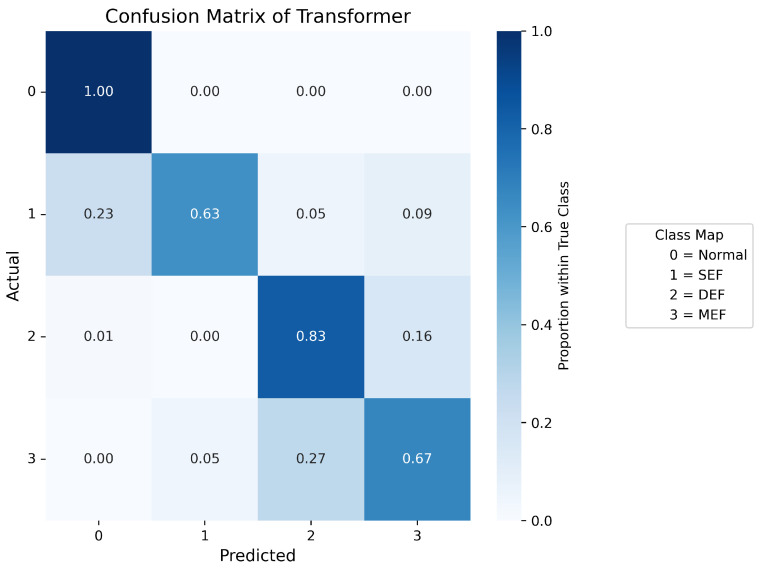
Confusion matrix of transformer.

Confusion matrices are used to display the probability distribution across each class and serve as a tool to evaluate the performance of a classification model by comparing its predicted outputs with the actual class labels. Figure 7, Figure 8, Figure 9, Figure 10 and Figure 11 present the confusion matrices of the different neural network models. All confusion matrices are evaluated on a test dataset containing 6565 samples.

Figure 8 shows the confusion matrix of the CNN–LSTM model, which achieves a high overall accuracy of approximately 98%, with predictions closely matching the true labels. Minor misclassifications remain in Label 1 (SEF) and Label 3 (MEF), accounting for about 2–3% of total errors, reflecting the similarity between the signal patterns of these two fault types. Figure 7 and Figure 9 present the confusion matrices of the CNN and Temporal Convolutional Network (TCN) models, respectively. Both achieve similar average accuracies of about 95%. The CNN model tends to misclassify Label 1 as Label 3, while the TCN model sometimes confuses Label 3 with Label 1. These errors arise from the close resemblance between the spatial–temporal characteristics of the two eccentricity faults. As both models rely heavily on convolutional operations, fine temporal transitions may be smoothed out, leading to reduced discrimination between SEF and MEF. Figure 10 illustrates the confusion matrix of the Deep Neural Network (DNN) model, which exhibits a higher rate of misclassification, particularly between Labels 1, 2, and 3. Around 40% of Label 1 samples are predicted as Label 3, and 16% of Label 3 samples are misclassified as Label 1. Additionally, 14% of Label 2 data are mistaken for Label 0. These results indicate that the DNN fails to capture complex temporal dependencies and relies mainly on static features, limiting its ability to distinguish dynamic fault conditions. The Transformer-based model, shown in Figure 11, achieves the lowest overall accuracy of approximately 80%. Although it performs well in recognizing normal conditions (Label 0), its accuracy for fault categories declines significantly (≈70%). Notable confusion exists between Labels 1 (SEF) and 3 (MEF), as well as between Labels 2 (DEF) and 3 (MEF). This pattern suggests that, given the limited window length and dataset size, the self-attention mechanism is less effective at capturing localized, periodic temporal variations in the current signals, resulting in weaker generalization across fault types.

From an overall perspective, the residual errors of the proposed CNN-LSTM model are highly concentrated between Label 1 (SEF) and Label 3 (MEF), while confusion with Label 0 (Normal) and Label 2 (DEF) is negligible. By inspecting the time-domain responses, it was observed that most misclassified windows occur near fault onset or during short transition periods, where the fault signatures are still evolving and the harmonic content is not yet fully separated. In addition, the finite window length, sensor filtering, and data acquisition pipeline introduce a small delay between the physical occurrence of the fault and the moment when its full signature is visible in the measured currents. In the proposed implementation, each diagnostic segment contains 4096 samples over a 20 ms window, so the effective detection delay introduced by the acquisition and processing chain remains small compared to the electrical period and the time scale of the mechanical dynamics. As a result, some early MEF windows retain SEF-like characteristics, and the CNN–LSTM occasionally assigns the dominant static component rather than the true mixed state.

Despite the promising performance of the tested models, certain limitations persist due to the complex temporal–spatial nature of motor current signals. CNN and TCN models tend to lose fine temporal details, causing confusion between similar fault types such as SEF and MEF. The DNN lacks sequence awareness, resulting in poor differentiation among dynamic faults, while the Transformer struggles with localized feature extraction and requires larger datasets to generalize effectively. Even the CNN–LSTM, though most accurate, exhibits a small but non-zero response delay and higher computational demands.

### 4.2. Performance Evaluation Under Different Hyperparameter Configurations

To ensure a fair comparison, all neural network models in the E-FDNet framework share a consistent training and validation strategy. The dataset is split into training, validation, and test subsets at the sample-identity level, so that segments from the same original recording do not appear in different subsets. Key hyperparameters (number of filters, kernel sizes, number of LSTM units, dropout rate, learning rate, and batch size) are tuned using a combination of manual trial-and-error and a coarse grid search. Each candidate configuration is trained on the training subset and evaluated on the validation subset with early stopping (patience = 20) and learning-rate reduction (factor = 0.5) when the validation loss plateaus.

To optimise the proposed E-FDNet, five different CNN–LSTM configurations were evaluated. The corresponding architectures are summarised in Table 6. CNN–LSTM Test 1 to Test 3 are designed to study the effect of architectural depth by varying the number and arrangement of convolutional, pooling, and LSTM layers. CNN–LSTM Test 1 uses a relatively shallow structure with a single convolutional block followed directly by an LSTM and dense layers. CNN–LSTM Test 2 introduces an additional convolutional block, while CNN–LSTM Test 3 further deepens the network by adding a third convolutional block and corresponding pooling operations.

CNN–LSTM Test 4 and Test 5 are then used to examine the influence of network width and training robustness. CNN–LSTM Test 4 shares the same macro-architecture and number of units as Test 2, providing an additional run to assess the stability of the training procedure under identical hyperparameter settings. In contrast, CNN–LSTM Test 5 increases the number of convolutional filters and LSTM units (Conv1D(64/128) and LSTM(128)), representing a wider and more complex variant of the Test 2 architecture.

The diagnostic performance of these configurations is reported in Table 7. CNN–LSTM Test 1, which employs the shallowest architecture, exhibits noticeably lower performance, with an F1-score of approximately 66.7%. In contrast, CNN–LSTM Test 2 and Test 3, which employ two and three convolutional blocks respectively, achieve substantially higher accuracies across all labels (approximately 94–96%). Among these, CNN–LSTM Test 2 provides the best trade-off between performance and architectural complexity and is therefore considered the primary candidate architecture.

For CNN–LSTM Test 4 and Test 5, the results show that the architecture used in Test 2 provides stable performance when retrained and that enlarging the network by increasing the number of filters and LSTM units does not yield a substantial improvement in diagnostic accuracy. Although Test 4 and Test 5 both achieve high F1-scores (94.3% and 94.9%, respectively), CNN–LSTM Test 2 consistently attains the highest overall F1-score (96.0%) and more balanced label-wise accuracies. Therefore, CNN–LSTM Test 2 is adopted as the final architecture for the proposed E-FDNet.

### 4.3. Performance Evaluation of the Proposed Motor Eccentricity Fault Diagnosis System Under Various Conditions

The diagnostic performance of E-FDNet was evaluated against two traditional methods—MCSA with PCA and Random Forest (RF) as the classifier, and MCSA with PCA and KNN as the classifier—as well as three baseline approaches: KNN [1], SVM combined with PCA [33], and a hybrid CNN + SVM + PCA method [14]. The speeds considered in Table 8 correspond to typical operating points of the PMSM drive and span low-, medium-, and high-speed regimes within the safe operating range of the test bench. These values also match the main operating conditions used in the experimental and PMM-based data collection, enabling us to evaluate the robustness of E-FDNet across representative practical speeds rather than at a single operating point.

Table 8 presents the comparative diagnostic performance of six methods—MCSA + PCA + KNN, MCSA + PCA + RF, SVM + PCA, KNN, CNN + SVM + PCA, and the proposed E-FDNet—evaluated at different rotational speeds (500 rpm–3000 rpm). The evaluation spans from 1500 to 3000 rpm (trained conditions) down to 500 and 1000 rpm (unseen conditions). At low speeds, the fundamental frequency of the current signals is reduced, so a longer time window is required for the distinct patterns of each operating condition to fully develop; this increases the similarity between classes in the time and frequency domains, especially in fault conditions. Under the trained conditions (1500–3000 rpm), both E-FDNet and the hybrid CNN + SVM + PCA achieve high accuracies (>98%), validating the efficacy of deep feature extraction. However, a divergence occurs in the unseen domain. The CNN + SVM + PCA model struggles to generalize at 500 rpm (dropping to 81.28%), and MCSA-based methods exhibit instability at 1000 rpm. Shallow models (KNN, SVM) perform poorly in these unseen regimes. Conversely, E-FDNet demonstrates remarkable domain generalization, maintaining a total accuracy above 94.6% even at 500 rpm. This confirms that E-FDNet does not merely memorize training data but learns robust, speed-invariant fault signatures (SEF, DEF, MEF), making it uniquely suitable for variable-speed applications where exhaustive training data for every speed is unavailable.

### 4.4. Response of the Proposed Motor Eccentricity Fault Diagnosis System

To validate the effectiveness of the proposed E-FDNet framework, comprehensive experiments were conducted under three fault scenarios: static eccentricity fault (SEF), dynamic eccentricity fault (DEF), and mixed eccentricity fault (MEF). In each case, the motor initially operated under normal conditions, after which fault signals were intentionally introduced.

**Figure 12:**
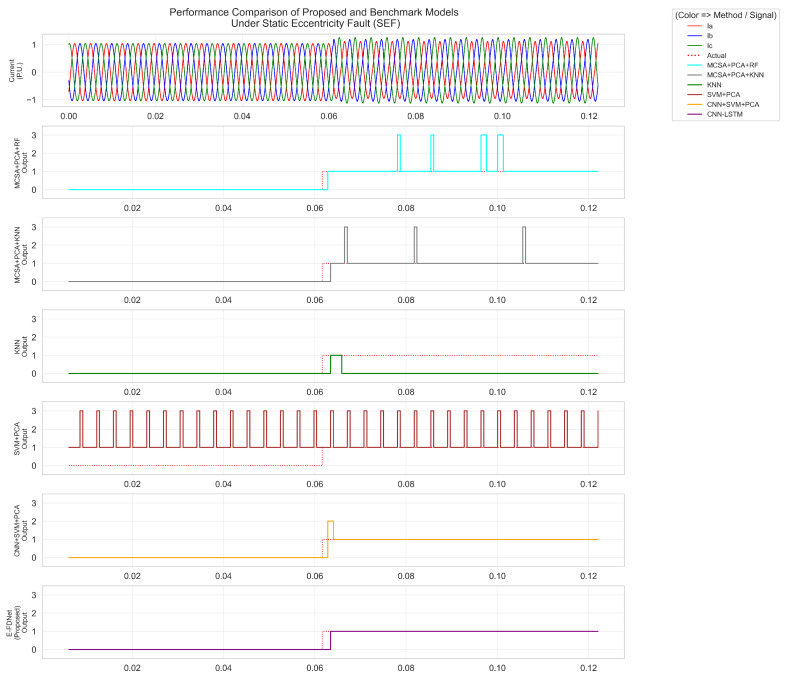
Comparison of model performance under static eccentricity conditions.

**Figure 13:**
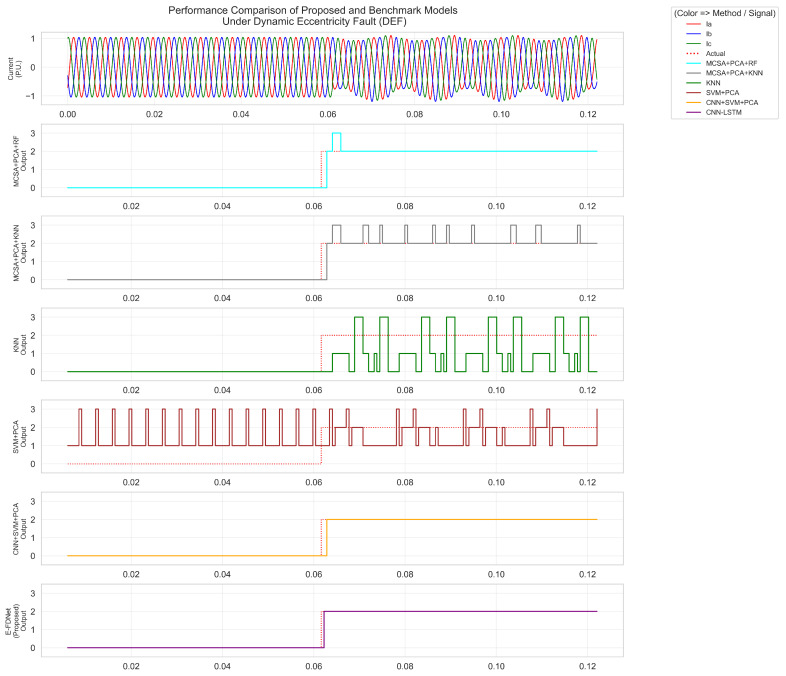
Comparison of model performance under dynamic eccentricity conditions.

**Figure 14:**
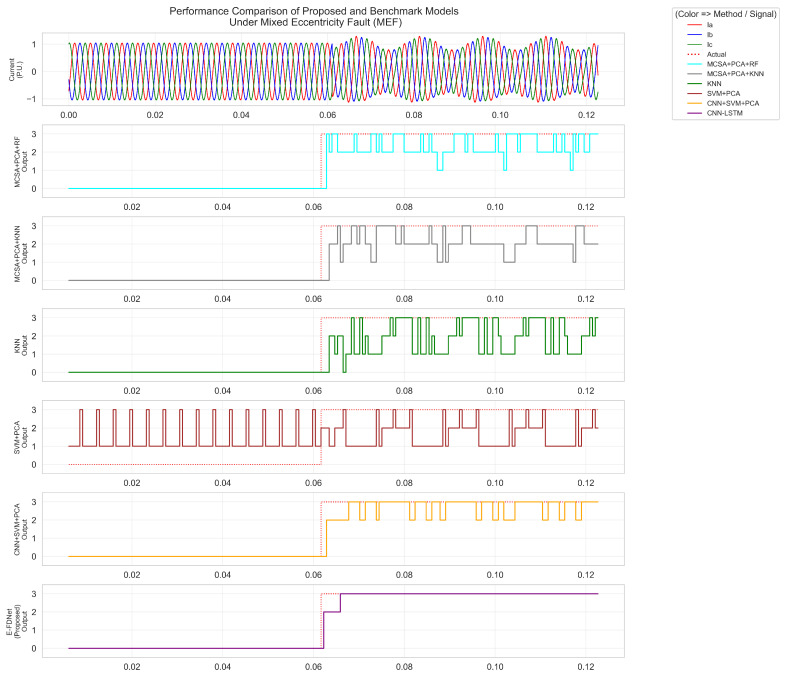
Comparison of model performance under mixed eccentricity conditions.

As shown in Figure 12, Figure 13 and Figure 14, label 0 represents the normal condition, label 1 corresponds to static eccentricity fault (SEF), label 2 to dynamic eccentricity fault (DEF), and label 3 to mixed eccentricity fault (MEF). Figure 12 presents the responses of different methods under SEF conditions. The fault was introduced at approximately 0.061 s. Both the MCSA + PCA + RF and MCSA + PCA + KNN methods exhibited similar responses, reacting around 0.062 s and labeling the condition as SEF. However, the RF- and KNN-based methods occasionally misclassified short segments as MEF. The KNN algorithm briefly labeled the condition as SEF at 0.062 s but quickly reverted to the normal label. The SVM + PCA method incorrectly labeled most of the signal as SEF, with multiple short periods mislabeled as MEF. The CNN+SVM + PCA model demonstrated improved accuracy, correctly detecting SEF after fault initiation, although it initially labeled a short segment as MEF before correcting to SEF. The proposed E-FDNet responded at 0.063 s, accurately identifying the SEF condition and maintaining a stable response throughout.

Figure 13 shows the responses under DEF conditions. The fault occurred at approximately 0.062 s. The MCSA + PCA + RF and MCSA + PCA + KNN methods again displayed similar behavior, both responding around 0.063 s. The RF-based method initially labeled the condition as DEF, then briefly as MEF before stabilizing back to DEF at about 0.070 s. The KNN-based method exhibited a similar pattern but with more frequent fluctuations toward MEF. The KNN approach performed poorly overall, misclassifying both normal and faulty periods, sometimes confusing SEF with DEF. The SVM + PCA method produced unstable outputs with continuous fluctuations. The CNN+SVM + PCA model improved the detection, responding at 0.064 s and maintaining better stability. In contrast, E-FDNet consistently recognized the normal region and transitioned accurately to DEF at 0.063 s, achieving a detection delay of approximately 0.001 s.

Figure 14 illustrates the responses under MEF conditions. The fault was introduced at approximately 0.062 s. Both MCSA + PCA + RF and MCSA + PCA + KNN methods produced similar results, responding around 0.063 s, but their outputs contained significant fluctuations across MEF, DEF, and SEF labels. The SVM + PCA model underperformed—it frequently mislabeled normal conditions as SEF and exhibited strong oscillations between MEF and DEF after the fault occurred. The CNN + SVM + PCA model detected the fault at 0.064 s, correctly recognizing the normal region but showing intermittent misclassifications between DEF and MEF. The proposed E-FDNet achieved the most stable and accurate performance, responding at 0.063 s. It initially detected DEF momentarily, then transitioned correctly to MEF at 0.068 s, maintaining stability thereafter.

Across all eccentricity fault scenarios, distinct differences in model behavior were observed. The MCSA-based approaches (PCA + RF and PCA + KNN) exhibited fast initial responses following fault onset but produced highly unstable outputs, frequently fluctuating between SEF, DEF, and MEF labels. Their classifications were inconsistent, with multiple short mislabeling periods before stabilizing, reflecting weak temporal robustness and delayed reliability. The SVM + PCA method performed poorly, labeling most regions incorrectly and showing continuous oscillations between fault states, indicating poor generalization and sensitivity to transient current variations.

The CNN + SVM + PCA hybrid model demonstrated moderate improvement, correctly detecting faults after onset and maintaining better alignment with actual transitions. However, it remained prone to transient misclassifications—especially during early fault stages—caused by noise and overlapping feature spaces among SEF, DEF, and MEF.

In contrast, the proposed E-FDNet achieved the most stable and accurate results in all cases. It correctly recognized normal regions and transitioned smoothly to the corresponding fault labels with minimal detection delay (approximately 0.001–0.003 s). Its outputs remained steady without oscillations throughout the fault duration, confirming its robustness and superior temporal–spatial feature extraction capability. These findings demonstrate that E-FDNet provides reliable, consistent, and real-time fault diagnosis performance for PMSM eccentricity detection compared with conventional and machine learning methods.

## 5. Conclusions

In conclusion, the proposed E-FDNet can identify three types of eccentricity fault (SEF, DEF, and MEF) within a single model and achieves the highest accuracy compared to the benchmark methods. Trained using both simulated and hardware-acquired data over a range of operating conditions, E-FDNet effectively discriminates between normal operation and faulty states. Experimental results confirm the model’s strong performance, achieving an average classification accuracy of approximately 98% and an F1-score of around 98% across all fault labels. Consistently outperforming traditional and deep-learning baselines, E-FDNet accurately distinguishes between normal and faulty conditions with minimal detection delays, typically ranging from 0.001 s to 0.003 s. Its stable and consistent outputs further underscore its robustness and reliability in diagnosing diverse eccentricity fault scenarios in a Motor Eccentricity Fault Diagnosis System (MEFDS).

Despite these strengths, several practical constraints remain, including the following: (i) suitable on-site deployment hardware is currently limited in availability and relatively expensive, which can hinder broad adoption—especially on edge or CPU-only platforms; (ii) inference latency scales with model size and input length, even when employing batching or basic quantization; and (iii) online or continual learning has not yet been evaluated and would require safeguards against catastrophic forgetting alongside rigorous validation under streaming updates. Although the proposed CNN–LSTM backbone has higher computational complexity than the classical baseline models, the measured end-to-end detection latency of E-FDNet remains within the 0.001–0.003 s range and is therefore compatible with real-time protection requirements for the PMSM drive. Future work will expand the training data to a wider range of fault severities and real-world variations, investigate model compression and quantization for cost- and resource-constrained hardware, optimize low-latency inference, and explore principled continual-learning strategies to further enhance adaptability, generalization, and diagnostic precision.

## Figures and Tables

**Table 1:** Specification of Mitsubishi HC-KFS43 Motor [17].

Variable (Unit)	Value
Input AC Voltage (V)	100–120
Rated Current (A)	2.3
Maximum Current (A)	6.9
Rated Power (kW)	0.4
Rated Speed (rpm)	3000
Maximum Speed (rpm)	4500
No. of Poles Pairs	4
Moment of Inertia (kg.cm^2^)	0.46
Rated Torque (Nm)	1.3
Maximum Torque (Nm)	3.8

**Table 2:** Details of the data used to train the neural network and the label assigned to each state.

Condition	Type of Data			Labeling
	Experimental Data	Simulation Data (PMM)	Simulation Data (FEM)	State (Class)
Normal	Yes	Yes	Yes	0
Static Eccentricity Fault (SEF)	No	Yes	Yes	1
Dynamic Eccentricity Fault (DEF)	No	Yes	Yes	2
Mixed Eccentricity Fault (MEF)	No	Yes	Yes	3

**Table 3:** Motor and Simulation Parameters of the HC-KFS43 Servo Motor.

Component	Parameter	Value/Type
General	Motor type	3-phase PMSM (Servo)
	Rated power	0.4 kW
	Rated speed	3000 rpm
	Rated torque	1.3 N·m
	Number of poles	8 (4 pole pairs)
Stator	Outer diameter	80 mm (approx.)
	Bore diameter	40.6 mm
	Slots	12
	Winding type	Copper, 3-phase, star (Y)
	Turns per coil	22–28
	Slot fill factor	0.40
Rotor	Topology	IPM–spoke type
	Magnet type	XG196/96 (NdFeB)
	Magnet thickness	2.5–3.0 mm
	Magnet arc coverage	∼0.8 pole pitch
	Rotor outer diameter	40.0 mm
	Rotor back-iron	4.0 mm
	Shaft diameter	11 mm
Air-gap	Radial length	0.3 mm

**Table 4:** Configurations of Various Neural Network Models.

**Layer**	**CNN1D**	**CNN-LSTM (Proposed)**	**DNN**
I	Conv1D(64, 7, ReLu)	Conv1D(32, 5, ReLu)	Flatten
II	MaxPooling1D(2)	MaxPooling1D(2)	Dense(512, Relu)
III	Conv1D(128, 5, ReLu)	Conv1D(64, 5, ReLu)	Dropout(0.3)
IV	GlobalAveragePooling1D	MaxPooling1D(2)	Dense(256, Relu)
V	Dropout(0.3)	LSTM(64, tanh)	Dropout(0.3)
VI	Dense(128, ReLu)	Dropout(0.3)	Dense(128, Relu)
VII	Dense(4, SoftMax)	Dense(64, ReLu)	DropOut(0.2)
VIII	-	Dense(4, SoftMax)	Dense(4, SoftMax)
**Layer**	**TCN**	**Transformer**	
I	Conv1D(64, 5, ReLu)	Dense(64)	
II	Dropout(0.2)	PositionalEncoding(200, 64)	
III	Residual Add()	MultiHeadAttention(200, 64)	
IV	Conv1D(64, 5, ReLu)	Residual Add()	
V	Dropout(0.2)	LayerNormalization()	
VI	Residual Add()	Dense(128, ReLu)	
VII	Conv1D(64, 5, ReLu)	Dropout(0.1)	
VIII	Dropout(0.2)	Dense(64)	
IX	Residual Add()	Residual Add()	
X	GlobalAveragePooling1D()	LayerNormalization()	
XI	Dense(64, ReLu)	Repeat Layers	
		III to X	
XII	Dropout(0.3)	GlobalAveragePooling1D()	
XIII	Dense(4, SoftMax)	Dropout(0.3)	
XIV	-	Dense(128, Relu)	
XV	-	Dense(4, SoftMax)	

**Table 5:** Performance of Different Types of Neural Network Implement in Eccentricity Fault Diagnosis Network (E-FDNet).

Neural Networks’ Types	Accuracy in Each Label (%)				F1 Score (%)
	0	1	2	3	
CNN	100	89.05	100	90.45	95.31
CNN-LSTM	100	97.54	100	97.50	98.86
DNN	92.54	67.56	87.75	75.38	82.00
TCN	99.83	90.23	99.77	88.73	95.08
Transformer	91.75	75.02	76.90	70.28	80.09

**Table 6:** Candidate CNN–LSTM architectures for the proposed E-FDNet.

**Layer**	**CNN-LSTM Test 1**	**CNN-LSTM Test 2 (Proposed)**	**CNN-LSTM Test 3**
I	Conv1D(32, 5, ReLu)	Conv1D(32, 5, ReLu)	Conv1D(32, 5, ReLu)
II	MaxPooling1D(2)	MaxPooling1D(2)	MaxPooling1D(2)
III	LSTM(64, tanh)	Conv1D(64, 5, ReLu)	Conv1D(64, 5, ReLu)
IV	Dropout(0.3)	MaxPooling1D(2)	MaxPooling1D(2)
V	Dense(64, ReLu)	LSTM(64, tanh)	Conv1D(128, 5, ReLu)
VI	Dense(4, SoftMax)	Dropout(0.3)	MaxPooling1D(2)
VII	-	Dense(64, ReLu)	LSTM(64, tanh)
VIII	-	Dense(4, SoftMax)	Dropout(0.3)
IX	-	-	Dense(64, ReLu)
X	-	-	Dense(4, SoftMax)
**Layer**	**CNN-LSTM Test 4**	**CNN-LSTM Test 5**	
I	Conv1D(32, 5, ReLu)	Conv1D(64, 5, ReLu)	
II	MaxPooling1D(2)	MaxPooling1D(2)	
III	Conv1D(64, 5, ReLu)	Conv1D(128, 5, ReLu)	
IV	MaxPooling1D(2)	MaxPooling1D(2)	
V	LSTM(64, tanh)	LSTM(128, tanh)	
VI	Dropout(0.3)	Dropout(0.3)	
VII	Dense(64, ReLu)	Dense(64, ReLu)	
VIII	Dense(4, SoftMax)	Dense(4, SoftMax)	

**Table 7:** Performance of CNN–LSTM Configurations and Selection of the Proposed E-FDNet.

Neural Networks’ Types	Accuracy in Each Label (%)				F1 Score (%)
	0	1	2	3	
CNN-LSTM Test 1	85.9	32.0	57.1	58.0	66.7
CNN-LSTM Test 2 (Proposed)	97.1	95.9	95.4	94.4	96.0
CNN-LSTM Test 3	96.5	86.7	95.8	87.4	92.6
CNN-LSTM Test 4	96.2	93.8	93.2	91.9	94.3
CNN-LSTM Test 5	96.9	92.9	95.8	91.8	94.9

**Table 8:** Performance comparison of different methods at different speed.

**Speed = 500 rpm**						
**Condition**	**MCSA + PCA + KNN**	**MCSA + PCA + RF**	**SVM + PCA**	**KNN**	**CNN + SVM + PCA**	**E-FDNet**
Normal	97.3%	97.2%	33.6%	5.6%	97.9%	94.1%
SEF	85.7%	88.1%	12.0%	3.6%	36.2%	94.0%
DEF	96.4%	97.3%	40.8%	4.5%	97.1%	94.2%
MEF	80.0%	87.3%	8.8%	2.5%	65.6%	96.6%
**Total**	**91.7%**	**93.5%**	**22.7%**	**3.2%**	**81.28%**	**94.6%**
**Speed = 1000 rpm**						
**Condition**	**MCSA + PCA + KNN**	**MCSA + PCA + RF**	**SVM + PCA**	**KNN**	**CNN + SVM + PCA**	**E-FDNet**
Normal	72.2%	91.9%	7.2%	38.9%	97.2%	94.3%
SEF	0.0%	14.8%	0.0%	11.2%	97.0%	95.0%
DEF	0.7%	1.5%	33.0%	19.7%	94.1%	95.3%
MEF	54.0%	30.4%	0.0%	32.5%	98.0%	97.4%
**Total**	**50.5%**	**50.0%**	**21.0%**	**30.07%**	**96.7%**	**95.3%**
**Speed = 1500 rpm**						
**Condition**	**MCSA + PCA + KNN**	**MCSA + PCA + RF**	**SVM + PCA**	**KNN**	**CNN + SVM + PCA**	**E-FDNet**
Normal	97.3%	97.2%	7.2%	93.2%	98.1%	98.5%
SEF	91.2%	92.8%	42.9%	90.0%	98.8%	99.0%
DEF	90.0%	92.1%	31.3%	79.8%	97.1%	97.5%
MEF	89.7%	91.8%	13.5%	80.9%	98.7%	99.2%
**Total**	**93.2%**	**94.3%**	**26.8%**	**87.6%**	**98.2%**	**98.6%**
**Speed = 2000 rpm**						
**Condition**	**MCSA + PCA + KNN**	**MCSA + PCA + RF**	**SVM + PCA**	**KNN**	**CNN+SVM + PCA**	**E-FDNet**
Normal	97.3%	97.3%	15.4%	95.1%	98.1%	98.5%
SEF	91.4%	92.5%	42.9%	92.7%	98.8%	99.0%
DEF	89.8%	92.1%	32.3%	80.0%	97.1%	97.5%
MEF	89.7%	92.3%	13.6%	81.2%	98.7%	99.2%
**Total**	**93.2%**	**94.4%**	**28.7%**	**89.0%**	**98.2%**	**98.6%**
**Speed = 2500 rpm**						
**Condition**	**MCSA + PCA + KNN**	**MCSA + PCA + RF**	**SVM + PCA**	**KNN**	**CNN + SVM + PCA**	**E-FDNet**
Normal	96.5%	96.5%	20.6%	94.9%	97.7%	98.0%
SEF	91.1%	92.5%	43.7%	91.1%	98.5%	99.0%
DEF	89.2%	90.6%	34.9%	80.0%	96.6%	97.5%
MEF	90.2%	92.9%	14.2%	81.2%	98.7%	99.2%
**Total**	**92.4%**	**93.6%**	**31.3%**	**88.0%**	**97.9%**	**98.4%**
**Speed = 3000 rpm**						
**Condition**	**MCSA + PCA + KNN**	**MCSA + PCA + RF**	**SVM + PCA**	**KNN**	**CNN + SVM + PCA**	**E-FDNet**
Normal	96.8%	96.9%	21.1%	95.0%	97.7%	98.0%
SEF	91.1%	92.5%	43.7%	91.1%	98.5%	99.0%
DEF	89.6%	91.4%	34.9%	80.2%	96.6%	97.5%
MEF	90.2%	92.7%	14.2%	81.2%	98.7%	99.2%
**Total**	**92.6%**	**93.9%**	**31.4%**	**88.1%**	**97.9%**	**98.4%**

## Data Availability

The original contributions presented in this study are included in the article. Further inquiries can be directed to the corresponding author.
